# Delphi Study: Designing Training to Enable Visual Impairment Services to Promote Physical Activity

**DOI:** 10.3390/vision7010008

**Published:** 2023-01-22

**Authors:** Rosie K. Lindsay, Lee Smith, Peter M. Allen

**Affiliations:** 1 Vision and Hearing Sciences Research Centre, Anglia Ruskin University, Cambridge CB1 1PT, UK; 2 Centre for Health Performance and Wellbeing, Anglia Ruskin University, Cambridge CB1 1PT, UK

**Keywords:** exercise, health, visual impairment, blind, social care

## Abstract

Research suggests adults with visual impairment would increase their physical activity (PA) if they were advised to by a professional working in visual impairment services. However, there are no training programs which are targeted at enabling these professionals to promote PA. Therefore, this study aims to inform a UK-based training program which facilitates PA promotion within visual impairment services. A modified Delphi technique was used, consisting of a focus group and two rounds of surveys. The panel included 17 experts in round one, and 12 experts in round two. Consensus was defined as equal to or greater than 70% agreement. The panel agreed training should: educate professionals on PA benefits, injury prevention, and wellbeing, address myths associated with PA, address health and safety concerns, help professionals to find local PA opportunities, and include a networking session for professionals in visual impairment services and local PA providers. The panel agreed training should also target PA providers and volunteers for visual impairment services, and that training should be delivered online and in-person. In conclusion, training should provide professionals with the capability to promote PA and to establish stakeholder partnerships. The present findings can inform future research which tests the panel’s recommendations.

## 1. Introduction

Regular physical activity (PA) is essential to prevent and manage non-communicable diseases including type 2 diabetes, cardiovascular disease, stroke, colon cancer and breast cancer [1]. Regular PA also promotes good mental health and can be beneficial for symptoms of depression [2] and anxiety [3], as well as improving overall quality of life [4,5]. The United Kingdom (UK) Chief Medical Officers’ Guidelines recommend each week adults do 150 min of moderate PA, or 75 minutes of vigorous PA, or a combination of the two, and strengthening exercises on two days [6]. However, in the UK, approximately 34% of men and 42% of women do not meet PA guidelines [6] and this problem is particularly prolific among populations with visual impairments. Adults who have self-reported poor sight even whilst wearing glasses or contact lenses [7] and people who have sight loss which is severe enough to be diagnosed as visually impaired are twice as likely not to meet PA guidelines as sighted people [8]. Thus, to address inequalities in health arising from low PA levels among people with visual impairment, interventions are required to target this specific population group. 

Research suggests that the majority of UK adults with visual impairment would increase their PA if they were advised to by a professional who works in visual impairment services [9]. Therefore, a professional who works for a visual impairment service, defined as someone who is employed to provide emotional or practical support to people with visual impairment, (e.g. outreach workers, low-vision rehabilitation officers, family support workers, emotional support workers) could play a key role in advising and supporting people to become more active. In 2022, British Blind Sport and partners published a guide for rehabilitation workers supporting blind and partially sighted adults with PA [10]. The guide summarises the benefits of PA, advice on doing PA safely, and examples of exercises which could be taught to people with visual impairment. Offering training for professionals which builds upon the recommendations outlined in this guide has several advantages. For example, training could encourage interaction between the trainer and trainees, allowing trainees to discuss and clarify points they are concerned about. Training can also utilise tools such as role-playing scenarios and group discussions which may help some people understand the material better than the guide alone. Moreover, training could incorporate a broader range of topics, and can be structured as an ongoing professional development program. This would provide an opportunity for continuous learning and the ability to expand upon the foundational knowledge provided by the guide. However, to our knowledge, there are currently no UK-based training programs aimed at supporting professionals in visual impairment services to promote PA. 

Training can be effective at increasing the number of professionals who promote PA as part of their practice. For example, the Moving Health Care Professionals’ Project (MHPP) has provided 28,000 healthcare professionals with clinical champion training, a peer training network which supports professionals to create a culture of discussing PA with patients within their organisation. An evaluation of the training found 40% of trainees had more conversations about PA with patients post-training [11]. Previous studies have also found training on PA promotion was associated with increased PA promotion among allied and non-medical healthcare professionals [12]. In summary, training is required to equip professionals with the skills they need to promote PA effectively and to increase PA promotion within visual impairment services.

Previous research which have aimed to develop PA promotion training for health and social care professionals have used a Delphi design to inform the training program design and components [13,14,15]. The Delphi technique was originally developed with the aim of reaching consensus among a group of experts through the use of multiple questionnaires interspersed with feedback [16]. There are several advantages of using the Delphi approach. Firstly, Delphi responses are anonymous, which can reduce the risk of participants conforming to the opinions presented by dominant members of the panel [17,18]. Furthermore, the controlled feedback can reduce the influence of irrelevant communications within a group, or communications focused on individual interest which can distract from problem solving. Controlled feedback gives participants the ability to reflect on the responses and allows participants to provide further understanding of the problems or issues which need to be addressed [16]. Online Delphi studies can also allow multiple participants to engage with the process at a time and place which is convenient for them, as participants are able to respond within a specified time period rather than attend a scheduled meeting, which may be required for other consensus building techniques such as nominal group technique [19] or a consensus conference [20]. Therefore, the present study uses a Delphi study design to inform the development of a training program to help visual impairment service professionals in the UK to promote PA to people with visual impairment. 

## 2. Materials and Methods

**Figure 1:**
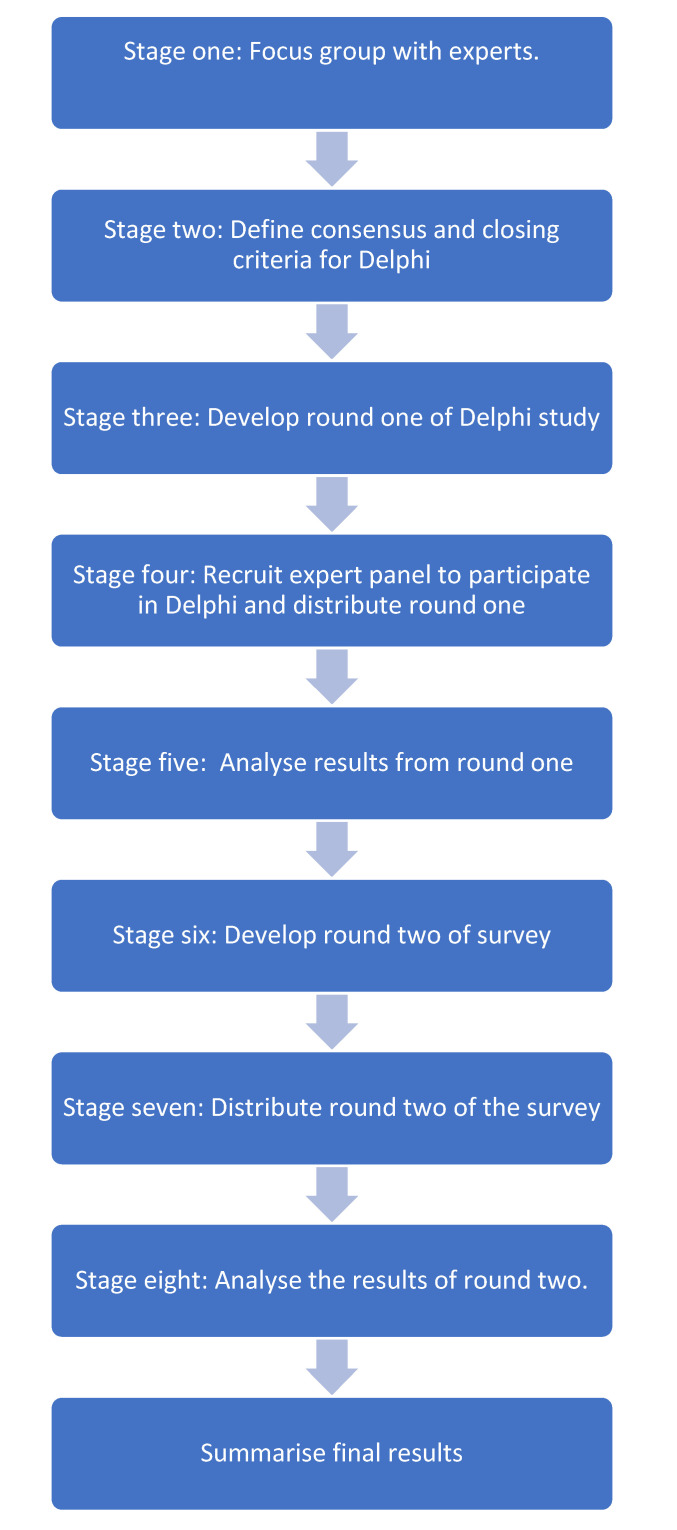
Modified Delphi process.

A modified Delphi method was used to reach consensus among experts. The main characteristic of a modified Delphi includes replacing the first round of survey questions with interviews or focus groups; they may also use fewer than three rounds of surveys [21]. Beyond the key defining features of a Delphi study there is no universally accepted method for conducting a Delphi, and thus the design can be adapted to meet the aims of individual studies. The research process is outlined in Figure 1. 

### 2.1. Stage One

Firstly, a focus group was held with four experts. The aim of this meeting was to gather input into what questions should be included in the Delphi to inform the development of a training program. Experts who had contributed to the development of the guide for UK rehabilitation workers supporting blind and partially sighted adults with PA produced in 2022 [10] were invited to participate via email. The experts were asked for their initial thoughts on the idea of a training program to help professionals in visual impairment services to advise and support people with their PA. In addition, the experts were provided with a list of potential ideas for the training program, which aimed to address barriers to PA promotion identified in previous interviews with professionals working in visual impairment services including: advice being inappropriate if the individual was not given time to grieve the loss of their sight, concerns about the appropriateness of advice, concerns regarding the health and mobility limitations of people who used their services, a lack of confidence to engage in PA among people who used their services, a lack of awareness of PA opportunities among people who used their services and environmental barriers. Experts were then asked for their opinions and if there were any additional components which should be included in a training program. Experts were also invited to provide any additional comments or insight which they felt may be important. During the focus group, two researchers recorded notes (RKL, PMA). After the meeting, the same two researchers compared and collated their notes to ensure that they had understood what was discussed and identified the key messages conveyed in the meeting. The key messages and discussion points raised in the focus group informed the development of round one of the Delphi. 

### 2.2. Stage Two: Defining Consensus and Closing Criteria

Prior to the start of the present study, it was decided that the study would be closed after two rounds, providing the panel had reached consensus on a range of components and design features, which could inform a feasible pilot trial of a training program. In the case that there were no components or design features which reached consensus the lead researcher planned to organise a consensus conference with panel members. 

Delphi studies vary with studies defining consensus from 50% to 100% agreement [22]. In the present study, consensus was defined as equal to or greater than 70% of participants agreeing that a specific component, or design feature, of the training should be included in the training program.

### 2.3. Stage Three: Developing Round One of the Delphi 

The aims of the survey administered in round one were to identify what components or design elements the expert panel agreed should be included in the training program, and to obtain further feedback and suggestions on components and design elements which should be included in the training program. The questions included single choice and multiple-choice questions, participants were also provided with free text boxes to add additional input and develop ideas. 

#### Question Content

In round one, participants were asked for feedback on the design and components of the training program. Participants were asked to select whether they thought the training should be delivered in person, online, or both. Participants were also asked what elements of a training program they thought should be included. The options included: ‘a section explaining the benefits of PA for people with visual impairment’, ‘a myth busting section which addresses common myths associated with PA’, ‘a practical role play activity where training course participants can practice giving motivational PA advice’, ‘show people how to find local PA opportunities’, ‘show people chair based activities they can pass on to service users’, ‘a peer support section where people can share their experiences and concerns about advising and supporting people with PA’, ‘a section addressing the health and safety concerns people may have about PA’. In addition, participants were given the option to add free text if they felt none of the options provided were applicable. 

To sustain long-term behaviour change among professionals, it was important that the training course encouraged participants to continue to develop their skills, and work on implementing PA promotion into their practice. Therefore, participants were asked what individuals or organisations should be encouraged to do post-training. The options included: ‘set up PA groups or experiences for staff’, ‘contribute three PA groups to the British Blind Sport activity finder’, ‘share an example of good practice which can be shared on social media and newsletters’, ‘present their experience of setting up PA groups/promoting PA to their service users at a bi-annual meeting with other visual impairment services, ‘complete a book or record of local groups/services that people can be sign posted to’, ‘produce a leaflet or newsletter which can be given/sent to service users about PA’, and ‘evaluate how many service users in their organisation engage in PA’. Participants were also given the option to add free text if they felt none of the options provided were applicable. Participants were also asked what rewards could be offered to people to ensure they engaged with the training and additional good practice after the training program had finished. Participants were provided with free text to input ideas. 

Finally, participants were provided with a free text option at the end of the survey to input any other ideas or feedback they may have which could inform the design of the training program. 

### 2.4. Stage Four: Recruiting the Panel for the Delphi Surveys 

Participants were eligible for inclusion in the Delphi panel if they were based in the UK and had one or more of the following areas of expertise or experiences: worked for a disability sport charity, worked for a visual impairment charity, provided emotional or practical support for people with visual impairment, ran or supported PA groups or services for people with visual impairment, provided or facilitated training for people who work in visual impairment services or had visual impairment themselves. The aim was to create a diverse panel of experts who could provide input in to either how to design training programs for professionals in visual impairment services, or how to facilitate PA among people with visual impairment. The email link was distributed to visual impairment charities and organisations via the mailing list of Visionary, a membership organisation for local visual impairment charities. The experts who had participated in the focus group also shared the link within their professional networks via email. Along with the invite to participate, participants received a link to the participant information sheet, which was followed by an informed consent form. All participants were required to provide informed consent prior to proceeding with the Delphi survey. Round one of the Delphi was open for responses for three weeks. 

### 2.5. Stage Five: Data Analysis 

All statistical analyses were conducted using SPSS Version 28. 0.0.0 (190). Descriptive statistics were used to summarise the population characteristics and the survey responses. In addition, the suggestions and feedback provided in round one were compiled into a list, which was then coded and grouped into categories using conventional content analysis [23]. The categories were used to inform the questions which were included in round two.

### 2.6. Stage Six: Developing Round Two of the Delphi

One week after round one had closed, participants were sent a summary of the findings in round one, alongside the questions for round two of the Delphi, via a link which was sent to their individual emails. 

#### 2.6.1. Who Should Be Included in the Training (Multiple Choice Questions)

In round one, several participants suggested the training should also target PA providers, volunteers and leisure facility providers; these suggestions were grouped as ‘training needs to target a wider audience’. To explore this suggestion further, the following question was included in round two: ‘should we target a broader range of people with the training program? Please select who you think the training should be targeted at in addition to professionals in visual impairment services.’ Participants could select: ‘Local physical activity providers’, ‘Visual impairment organisation’s volunteers’, ‘Physical activity group’s volunteers’, or ‘We should only target visual impairment service professionals’.

#### 2.6.2. Inclusion of a Networking Session (Single Choice Questions) 

Another reoccurring suggestion which came up in people’s responses to the round one survey, was that training should promote partnerships between visual impairment services and local PA providers. Therefore, participants were asked ‘do you think that we should include a networking session as part of the training, which allows local physical activity providers and visual impairment services to meet?’ Participants could select ‘yes’ or ‘no’, for participants who responded ‘yes’ a follow up question was included which asked ‘how should the networking sessions be delivered?’, participants could select ‘online’, ‘in-person’ or ‘both’.

#### 2.6.3. Managing PA Effectively (Multiple Choice Question)

Participants also made suggestions in round one that training should include information about how to manage PA and recovery. Therefore, participants were asked to select which of the following components they thought should be included in a training program: ‘how and when to refer to a physiotherapist’, ‘injury prevention training’, ‘information about recovery strategies’, ‘a wellbeing section, e.g., meditation/relaxation’ or ‘none of the above’.

#### 2.6.4. Additional Ideas for a Training Program (Multiple Choice Question) 

Participants’ additional suggestions in round one were compiled into a list of options, and participants were asked to select which options they thought should be included in the training program. Participants were asked to select a maximum of three to ensure that the training program developed was based on what participants thought should be prioritised in a training program, and to ensure the training program could be feasibly delivered across the UK within the context of time and resource constraints. Participants could select from the following options: ‘improve awareness of local charities who provide PA activities’, ‘improve awareness of available adapted equipment’, ‘how to develop a list of local accessible sessions in the community’, ‘develop knowledge of barriers faced by visually impaired people and solutions’, ‘how to source grants’, ‘how to do a risk assessment’, ‘give people ideas for group exercise or partnered exercise’, ‘do mystery shopping-type exercises as ways to ensure appropriate support is being offered and educate exercise providers if necessary’, ‘a section with ideas on how to be person-centered with your support’, ‘a section on how to do remote exercise safely (e.g., phone or video based exercises)’, ‘refresher training to update skills’, ‘find and train local physical activity champions within organisations’, ‘share feel good stories when someone has changed their life through exercise’, ‘include Sim specs (glasses which simulate different types of visual impairments), so staff can experience visual impairment and therefore provide better support’, ‘delivery by someone with lived experience of being blind/partially sighted so they can talk about real life experiences (challenges and solutions)’, ‘a section on how to account for cultural sensitivities’ or ‘none of the above’.

#### 2.6.5. Incentives (Multiple Choice Questions)

In round one, participants provided suggestions of incentives that could be offered to encourage professional in visual impairment services to participate in training. The suggestions were compiled into a list, and in round two, participants were asked to select all of the options which they thought would incentivise visual impairment service professionals to participate. The following options were provided: ‘vouchers for sports shops’, ‘connections with sports organisations, e.g., local gyms/sports/venues/coaches, so that establishing groups is easier’, ‘any funding towards completion of training’, ‘resources/links to facilitate PA’, ‘free membership to activities’, ‘certificate of achievement’, ‘Continued Professional Development (CPD) points’, ‘accreditation, recognition, e.g., gold, silver, bronze standards’, ‘a course handbook with ideas of exercises and tips on how to make them accessible’, ‘some free sessions at a local leisure centre for participants’ or ‘no rewards/incentives should be offered’.

In round one, participants also provided suggestions of incentives that could be offered to encourage PA providers to participate in training. The suggestions were compiled into a list, and in round two, participants were asked to select all the options which they thought could incentivise PA providers to participate. The following options were provided: ‘a badge they can display showing staff have visual impairment awareness’, ‘a course handbook with ideas of exercises and tips on how to make them accessible’, ‘PA sessions to encourage good practice’ or ‘no rewards/incentives should be offered’.

### 2.7. Stage Seven: Distributing Round Two of the Delphi

The survey was sent to all respondents in round one via a link sent to their individual emails. To ensure that the response rate reached a minimum of 70%, as recommended to ensure the Delphi process is rigorous [24], a follow up email was sent one week prior to the survey closing. Round two of the Delphi was open for responses for three weeks. 

### 2.8. Stage Eight: Data Analysis 

All statistical analyses were conducted using SPSS Version 28. 0.0.0 (190) (IBM, Armonk, NY, USA). Descriptive statistics were used to summarise the population characteristics and the survey responses. 

## 3. Results

Seventeen participants responded to round one, and twelve participants responded to round two (70.6% rate). Table 1 presents the distribution of expertise of the panel in rounds one and two; although the panel size decreased in round two, the range and distribution of expertise of the respondents in both rounds was similar. Table 2 presents the areas in which consensus was achieved in round one, and Table 3 presents the areas in which consensus was achieved in round two. 

It is also important to highlight several training program components did not meet consensus in round one; however, the majority of participants agreed these components should be included in a training program. For example, most participants agreed there should be a section which teaches professionals chair-based exercises they can then in turn teach to people who use their services (64.7%), and a peer support section where people can share their experiences and concerns about advising and supporting people with PA (64.7%). In summary, the components which reached consensus could be considered as core components of a training program; however, other components should not be discounted and could be part of further training and professional development.

In round two, although ‘injury prevention training’ and a ‘wellbeing section’ were the only additional components which over 70% of the participants agreed should be in the training program, most participants reported ‘how and when to refer to a physiotherapist’ (58.3%) and ‘information about recovery strategies’ (66.7%) should also be included in the training program. These findings indicate that a key focus of the training program should be on supporting professionals to minimise any risks associated with PA for their service users, and different professionals may require different support with varying aspects of health and safety. In terms of incentives, over 70% of participants reported ‘connections with sports organisations’ could incentivise professionals to participate in the training. However, none of the participants agreed that funding for those who complete the training should be offered as an incentive. This could reflect that participants felt professionals should be intrinsically motivated to engage in the training program. For example, in round one, one participant stated, “I would hope that the benefits of completing the training ‘sells itself’ and, therefore, individuals and organisations take part for the right reasons (and not because of rewards/incentives)”. Similarly, another participant stated that to incentivise professionals to participate in training it is important to “make it easy, fun and engaging”. On the other hand, all participants agreed that at least one type of incentive should be offered to encourage professionals in visual impairment services and PA providers to engage in the training program. In summary, offering incentives may be important for initially engaging professionals in the training; however, the results suggest offering incentives which utilise intrinsic motivation may be more important than offering extrinsic motivators such as funding. 

## 4. Discussion

The aim of this Delphi study was to reach expert consensus on design elements and training program components, which could inform the development of training to help professionals in visual impairment services to promote PA. Based on the consensus reached in the Delphi, several recommendations can be made. Firstly, training should target a broader remit of people to enable training to be effective, including local PA providers and visual impairment service volunteers. In addition, a networking session which allows visual impairment services and local PA providers to meet should be part of the training. Both training and the networking session should be delivered online and in-person. The components which should be prioritised in the training include educating professionals about the benefits of PA, addressing common myths associated with PA, showing people how to find local PA opportunities, injury prevention training, education about wellbeing, e.g., meditation/relaxation and addressing health and safety concerns about PA. To incentivise people to attend, the training should advertise that participating will help them to build connections with PA organisations, e.g., local gyms/sports/venues/coaches so that establishing PA groups for people with visual impairment is easier for them. To encourage sustained behaviour change post-training, the training should encourage attendees to share examples of good practice, which can be shared on social media and in newsletters.

The findings of the present study highlight the importance of developing relationships between visual impairment services and PA providers. Organising workshops and training which promote cross-sector collaboration has also been identified as a facilitator for social prescribing initiatives [25]. Previous literature suggests that to develop positive relationships between sectors, networking and training sessions should aim to: create shared understanding and attitudes across different sectors, share best practice, discuss processes, react to challenges, facilitate feedback between sectors, establish effective communications, establish how the sectors will be managed and led, and agree on steps to implementation [26]. In summary, the training and networking session will need to be carefully managed to ensure that different sectors understand their responsibilities when working together, and the processes required to work together effectively. 

Experts also agreed that training and networking should be available both in-person and online. Providing in-person and online training could provide benefits for potential attendees. Firstly, offering training online may make the training accessible to people for whom travel to a venue is a barrier. In addition, training delivered online could be recorded which will allow people who are not able to attend in real time to learn from the training. On the other hand, offering training in person may also encourage attendees for whom technology is a barrier to accessing training [26]. Previous studies have reported that blended learning may also improve knowledge compared to in-person only teaching [27,28,29,30], thus offering people content online and in-person may also improve training outcomes. However, it is important training and networking delivered online and in-person incorporates elements which encourage interaction amongst people engaging with the training online and in-person. Furthermore, it is important that the individual delivering the training or networking session focuses on including people who have attended online, to ensure they receive the same experience as those who have attended in-person [31]. Overall, providing training and networking in-person and online could improve training accessibility and outcome; however, the contents and delivery needs to be tailored to allow all attendees to benefit equally. 

The Delphi also identified that the training should include components which address health and safety concerns and provide advice on injury prevention. It is important that concerns about the safety of sport for people with visual impairment is not a barrier to participation. Therefore, these components should aim to reassure people that the benefits of PA outweigh the risks of being inactive [32], rather than compounding the safety concerns attendees of the training may already have. For example, people with visual impairment have a higher risk of falling than sighted populations [33], therefore, training should highlight the importance of reducing environmental and personal trip hazards. However, training should also emphasise that PA can be a mechanism which reduces the risk of falling, as PA can improve visual cognition [34] and balance [35,36]. Furthermore, strength training can reduce sports injuries to less than one third [37], therefore, the training should encourage attendees to promote strength training as part of PA promotion. In summary, it is important that training helps attendees to minimise the risk of PA for people with visual impairment and reassures attendees that PA can be beneficial and safe for people with visual impairment. 

Although the Delphi provided valuable insight in to how to design a training program, the findings should be considered in light of the study limitations. Firstly, the panel consisted of 17 participants in round one, and 12 in round two. The small sample size and high dropout rate in round two may limit the generalisability of these findings, however a sample of 12 is deemed to be sufficient for achieving consensus in a Delphi study [38]. Future studies using larger sample sizes should also consider collecting demographic data from participants such as age, gender and ethnicity to ensure that the components of the Delphi which reach consensus are representative of a diverse population, rather than a particular sociodemographic group. Furthermore, the present study aimed to inform a UK-based training program, and recruited participants from the UK, thus, the results of this research may need to be replicated in different countries in order to inform training programs that are context-specific. In addition, the expert consensus cannot be considered fact [39] and opinions held by the minority may be marginalised. Therefore, it is important that once the training program is developed further, evaluation and feedback is sought, to ensure the training is effective and refined if necessary.

## 5. Conclusions

The present Delphi identified design elements and components of training which reached consensus among an expert panel. Overall, the Delphi highlighted the need to target a broader audience with a training program, and to include a range of training program components which address barriers to PA promotion such as lack of knowledge about PA, and health and safety concerns. These findings can be used to inform further research, which can then be evaluated to inform future larger scale training programs. 

## Figures and Tables

**Table 1:** Areas of expertise of the panel in round one.

Areas of Expertise of the Panel	Round One N (%)	Round Two N (%)
Work for a disability sport or visual impairment charity	13 (76.5%)	10 (83.3%)
Provides emotional or practical support for people with visual impairment	12 (70.6%)	10 (83.3%)
Runs or supports visual impairment specific physical activity groups or services	7 (41.2%)	5 (41.6%)
Provides or facilitates training for people who work in visual impairment services	7 (41.2%)	5 (41.6%)
Provides emotional or practical support for people who are deafblind and have multiple disabilities	1 (5.8%)	1 (8.3%)
Visually impaired themselves (diagnosed as severely sight impaired)	4 (23.5%)	3 (25%)
Visually impaired themselves (diagnosed as sight impaired)	3 (17.6%)	2 (16.6%)

**Table 2:** Components which reached consensus in round one.

Category	Components Which Reached Consensus (% of Sample Who Agreed That the Component/Design Feature Should Be Included)
How to deliver training	Training should be delivered both online and in person. (82.4%)
Training program contents	A section explaining the benefits of physical activity for people with visual impairment. (70.6%) A myth busting section which addresses common myths associated with physical activity (e.g., physical activity is not safe for older adults, exercise is not safe for people with chronic conditions, exercise is not safe for people with a visual impairment). (76.5%) Show people how to find local physical activity opportunities on the activity finder provided by British Blind Sport. (70.6%) A section addressing the health and safety concerns that people may have about physical activity. (82.4%)
Post-training components	To encourage people to continue to promote physical activity as part of their practice once the training has ended individuals and organisations should share examples of good practice which can be used to share on social media and newsletters. (76.5%)

**Table 3:** Components which reached consensus in round two.

Category	Components Which Reached Consensus (% of Sample Who Agreed That the Component/Design Feature Should Be Included)
Who the training should target in addition to visual impairment service professionals	Local physical activity providers (100%) Visual impairment service’s volunteers (75%)
Networking	Agreed that a networking session which allowed physical activity providers and visual impairment services to meet should be included (100%)
Mode of delivery	Agreed that a networking session should be held both online and in-person (100%)
Additional components which should be included to help people manage physical activity and recover from physical activity	Injury prevention training (83.3%) A wellbeing section, e.g., (meditation/relaxation) (83.3%)
Incentives	Connections with sports organisations, e.g., local gyms/sports/venues/coaches so that establishing groups is easier (75%)

## Data Availability

The data presented in this study are available on request from the corresponding author.
